# Long-term work retention after treatment for cancer: a systematic review and meta-analysis

**DOI:** 10.1007/s11764-020-00862-2

**Published:** 2020-03-11

**Authors:** Angela GEM de Boer, Steffen Torp, Adela Popa, Trine Horsboel, Vesna Zadnik, Yakir Rottenberg, Edit Bardi, Ute Bultmann, Linda Sharp

**Affiliations:** 1 Coronel Institute of Occupational Health, Amsterdam Public Health Research Institute, Amsterdam UMC, University of Amsterdam, Amsterdam, The Netherlands; 2 Department of Health, Social & Welfare Studies, University College of South-Eastern Norway, Notodden, Norway; 3 Lucian Blaga University of Sibiu, Sibiu, Romania; 4 The Danish Cancer Society Research Center, Copenhagen, Denmark; 5 Institute of Oncology Ljubljana, Ljubljana, Slovenia; 6 Sharett Institute of Oncology, Hadassah-Hebrew University Medical Center, Jerusalem, Israel; 7 Kepler Universitäts Klinikum, Linz, Austria; 8 University Medical Center Groningen, University of Groningen, Groningen, the Netherlands; 9 Population Health Sciences Institute, Newcastle University Centre for Cancer, Newcastle University, Newcastle upon Tyne, United Kingdom

**Keywords:** Cancer, Work retention, Employment, Work ability, Return-to-work, Longitudinal studies, Prospective studies, Meta-analysis

## Abstract

**Purpose:**

Almost half of people diagnosed with cancer are working age. Survivors have increased risk of unemployment, but little is known about long-term work retention. This systematic review and meta-analysis assessed work retention and associated factors in long-term cancer survivors.

**Methods:**

We searched Medline/Pubmed, Embase, PsychINFO, and CINAHL for studies published 01/01/2000–08/01/2019 reporting work retention in adult cancer survivors ≥ 2 years post-diagnosis. Survivors had to be in paid work at diagnosis. Pooled prevalence of long-term work retention was estimated. Factors associated with work retention from multivariate analysis were synthesized.

**Results:**

Twenty-nine articles, reporting 21 studies/datasets including 14,207 cancer survivors, were eligible. Work retention was assessed 2–14 years post-diagnosis. Fourteen studies were cross-sectional, five were prospective, and two contained both cross-sectional and prospective elements. No studies were scored as high quality. The pooled estimate of prevalence of long-term work retention in cancer survivors working at diagnosis was 0.73 (95%CI 0.69–0.77). The proportion working at 2–2.9 years was 0.72; at 3–3.9 years 0.80; at 4–4.9 years 0.75; at 5–5.9 years 0.74; and 6+ years 0.65. Pooled estimates did not differ by cancer site, geographical area, or study design. Seven studies assessed prognostic factors for work retention: older age, receiving chemotherapy, negative health outcomes, and lack of work adjustments were associated with not working.

**Conclusion:**

Almost three-quarters of long-term cancer survivors working at diagnosis retain work.

**Implications for Cancer Survivors:**

These findings are pertinent for guidelines on cancer survivorship care. Professionals could focus support on survivors most likely to have poor long-term work outcomes.

**Electronic supplementary material:**

The online version of this article (10.1007/s11764-020-00862-2) contains supplementary material, which is available to authorized users.

## Introduction

The sustained improvements in detection and treatment of many types of cancer have steadily increased life expectancy after cancer treatment [1]. During the next decade, a further rapid increase in the number of new cancer diagnoses in the population and a growing number of cancer survivors are expected [1].

Almost half of the people diagnosed with cancer are of working age [2] and it is therefore likely that the prevalence of cancer survivors in the work force will increase. In addition, the retirement age is rising in many countries, implying that more cancer survivors will be part of the working population [3].

For both cancer survivors themselves and society, returning to work is important. Survivors often regard returning to work as regaining normality and self-respect [4]. It contributes to their quality of life [5] and provides them with financial security [6]. From the viewpoint of the ageing society, it is an economic and social necessity to encourage survivors to return to work whenever possible [7].

Cancer survivorship is associated with a range of enduring physical and psychological effects including long-lasting fatigue [8, 9], depression [9, 10], physical complaints [9, 11], and neurocognitive limitations [9, 12, 13]. These long-term outcomes of cancer treatment can have persistent impact on the work ability of survivors [14]. As a result, cancer survivors have been shown to have an increased risk of unemployment compared to the general population in long-term follow-up studies [15–17].

Several reviews on the impact of cancer treatment on short-term work outcomes have been published [18–20]. These reviews showed return to work rates between 39 and 93% within 1–2 years after diagnosis. However, the employment pathways of cancer survivors could change after this point because treatment for cancer can, increasingly, be a long process (taking a year or more) and survivors can have persistent long-term effects which may last well beyond 2-year post-diagnosis [21]. However, the long-term effects of cancer treatment on work outcomes have not been systematically reviewed. In addition, the influence of prognostic factors on long-term work outcomes has not been synthesized.

A systematic review on the long-term work status of cancer survivors would be of value both for helping shape expectations of new cancer patients regarding likely long-term outcomes (including work outcomes), and in psychosocial survivorship care, when counselling survivors on the long-term psycho-oncological outcomes after treatment [22]. This type of information can therefore help improve survivors’ quality of life by preventing work loss and distress.

The aims of the current study are therefore (i) to systematically assess long-term work retention among cancer survivors 2 years and more after diagnosis and (ii) to assess associated factors for work retention in long-term cancer survivors.

## Materials and methods

### Search strategy

We followed the Preferred Reporting Items for Systematic Reviews and Meta-Analyses (PRISMA) guidelines in conducting this review and preparing the manuscript [23]. We searched four databases (Medline [Pubmed], Embase, PsychINFO, CINAHL) to identify studies reporting workforce retention in long-term cancer survivors, published from 01/01/2000 to 08/01/2019. We defined long-term survivors as those who were at least 2 years from diagnosis [24]. Combinations of disease-related, work-related, and survivor-related search terms were used (Supplementary Table S1). Disease-related terms included *cancer*, *neoplasm*, *carcinoma*, *tumour*, *oncology*, *radiotherapy* and *chemotherapy*; work-related terms included *employment*, *unemployment*, *retirement*, *sick leave*, *sickness absence*, *absenteeism*, *presenteeism*, *work*, *occupation*, *work ability*, *work disability*, *disability management*, *rehabilitation* and *vocational*; and survivor-related terms included *survivor*, *survival*, and *long-term.* Wildcards and alternative spellings were used where appropriate. Only full papers published in peer-reviewed journals were eligible; we did not include conference abstracts or the gray literature, the former because abstracts rarely contain sufficient detail to be able to determine eligibility (or appraise quality) and the latter because such studies are difficult to identify systematically. Reference lists from reviews of cancer and work identified in the electronic searches and of eligible papers were scrutinized to identify any potentially eligible articles which might have been missed by the electronic searches.

### Eligibility criteria

To be included, papers had to include survivors who were all at least 2-years post-diagnosis. If study participants were a range of times from diagnosis (e.g., 6 months to 3 years), then the group of long-term survivors (at least 2-years post-diagnosis) had to be reported separately. Studies were eligible if they included cancers at any site (invasive or in situ) diagnosed in adults (defined as those aged 18 and older); studies of cancers diagnosed in children or adolescents were excluded as their employment outcomes may differ from those of survivors diagnosed in adulthood. All survivors in the studies needed to be employed or in paid work at the time of diagnosis (either for an employer or self-employed); studies where survivors were in the labor market at diagnosis but were not all employed/working (e.g., some were unemployed or job seeking), and the group employed/working were not reported separately, were not eligible. In terms of outcomes, studies had to report a measure of work retention (e.g., percentage employed/unemployed/working or percentage return to work) at 2-years post-diagnosis and/or later time-points. Our primary interest was in the proportion of survivors who were working long term; therefore, if a study reported survivors by work status category (e.g., on sick leave, retired, working) or multiple measures of work retention, we abstracted the figure for those who were working (i.e., back in the workplace) at the time the outcome was assessed. Studies which reported raw figures such as numbers or percentages of work retention were included and those reporting only odds ratios or relative risks were excluded. Quantitative cross-sectional or prospective observational studies, with or without a control/comparison groups, were eligible, as were observational studies nested within randomized controlled trials. Trials of vocational or rehabilitation interventions were excluded as the return to work experiences of participants may not have been typical of those of the base population. In addition, we excluded studies of survivors of occupational cancers because their return to work experiences may not be typical of all survivors. Only studies where the base population was known were included. To have a reasonable degree of precision in the estimates of work retention, we excluded studies where outcome data was available on less than 50 individuals. No language limits were placed on the search.

### Data extraction

Two of the authors independently screened each title and abstract. Full text of papers considered potentially eligible for inclusion by either or both reviewers were read independently by the same two reviewers and their suitability for inclusion assessed. The reviewers then compared results and discussed any discrepancies; a third author (AdB or LS) was called on in the event of disagreement. When uncertainty about eligibility remained, authors of papers were contacted; if they did not respond after a reasonable time, the paper was excluded.

Data abstraction from eligible papers was done independently by two authors. Information extracted on study characteristics included (1) study location (country); (2) study design; (3) study population including diagnosis, sex, age; (4) time-points at which outcomes were assessed; and (6) which outcome(s) were assessed and how. Information was abstracted on work retention (preferentially percentage working or returned to work; percentage employed otherwise; percentage unemployed was converted into percentage employed). Information was also abstracted on any risk factors for work retention (categorized as patient-related, clinical or work-related) considered. If multivariable analyses of risk factors were reported, results of these were abstracted and reported. Finally, details of any other work-related outcomes reported (e.g., income, working hours) were extracted.

### Analysis

Meta-analysis was conducted in Stata 15 [College Station, Texas, USA], using the *metaprop_one* command [25], fitting a logistic-normal random effects model with inverse-variance weights and the Freeman-Tukey double arcsine transformation. The pooled proportion working or employed was computed across all studies. Results for studies which reported the inverse of the outcome of interest (e.g., unable to work) were subtracted from 100 before inclusion. In the primary analysis, for studies which reported multiple time-points, results from the earliest time-point post-diagnosis were used; a sensitivity analysis was conducted in which results for the last time-point post-diagnosis were used instead. Pooled proportions were also computed for a range of time windows post-diagnosis: 2–2.9 years, 3–3.9 years, 4–4.9 years, 5–5.9 years, and 6+ years. In these analyses, studies which reported results at multiple time-points were included in the analysis for each relevant time-point. Subgroup analysis was performed for cancer site, geographical area as defined by the World Health Organization [26], study design, and sampling frame for the cancer population. All tests of statistical significance were two-sided.

### Quality appraisal

Full papers of eligible studies were critically appraised, by two authors (AdB, LS), using the Methodological Index for Non-Randomised Studies (MINORS) [27]. Where multiple papers were available from the same study/using the same dataset, we appraised the paper which included data from a comparative group or, failing that, the earliest published paper. Studies were scored on 12 items, eight of which applied to all studies: the aim of the study, inclusion and retention rate, prospective data collection, employment endpoints, unbiased assessment of endpoints, follow-up time after diagnosis, loss to follow-up, and prospective calculation of sample size. Four additional items applied only to those studies with control/comparison groups: comparable control group, contemporary control group, baseline equivalence of groups, and adequate statistical analysis. Each study was scored 0/1/2 for each item. Thus, the total possible score for a non-comparative study was 16 and for a comparative study was 24. High quality was considered a score of ≥ 16/24 [2].

## Results

### Study selection

**Fig. 1:**
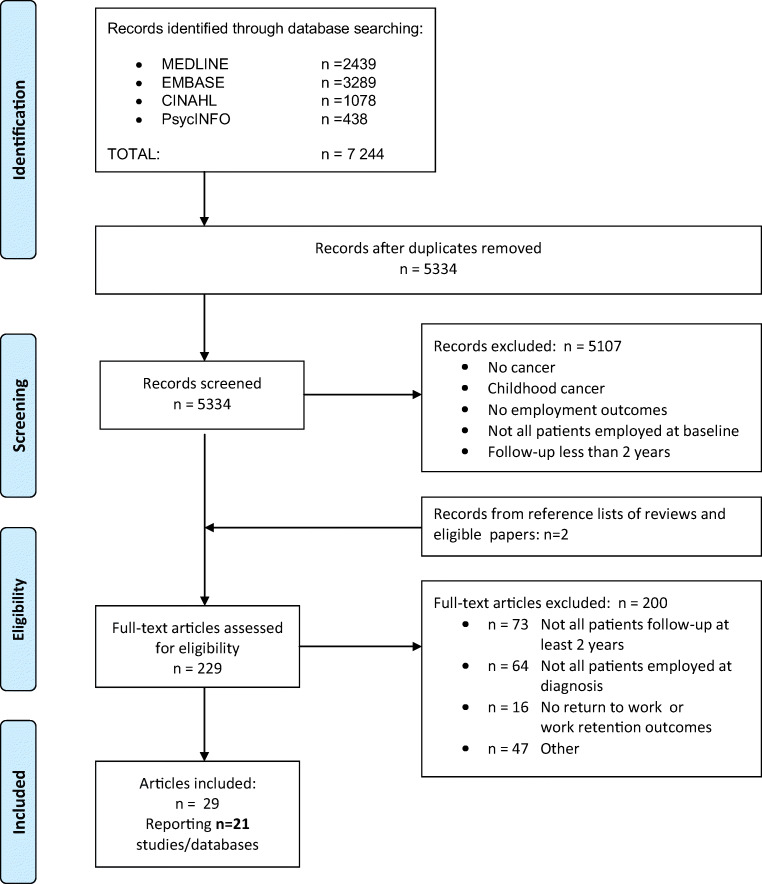
Flow diagram of included studies

Figure 1 shows the number of papers identified, screened, and included. After removal of duplicates, the initial searches yielded 5334 records. After screening of titles and abstracts, 229 articles remained. Following full-text review of these, 29 articles were deemed eligible. These final 29 articles reported findings from 21 different studies or datasets (Table 1) [28–56].

**Table 1:** Characteristics of eligible studies and prevalence of work retention among longer-term cancer survivors

Author and year	Country, state	Study design and source of patients	Study population	Time-point(s) outcome assessed^a^	Outcome	Results
Amir et al., 2007 [28]	UK, England	Cross-sectional survey Source: Population-based cancer registry	*N* = 267; 48% breast, 14% colorectal, 9% prostate, 6% lung, 23% other; 73% female; mean 48 years	3 years	Working; Self-reported postal questionnaire	82% working
Blinder et al., 2012; [29] Blinder et al., 2013 [30]	USA, California	Prospective survey Source: breast cancer treatment	*N* = 290 and 274; 100% breast cancer; 100% female; median 49 years	3 and 5 years	Working/return to work; self-reported by telephone interview	3 years: 56% working 5 years: 72% returned to work
Bradley and Bednarek, 2002; [31] Bednarek and Bradley, 2005 [32]	USA, Michigan	Cross-sectional survey Source: Population-based cancer registry	*N* = 141; 29% breast, 21% colorectal, 23% lung, 27% prostate; 47% female; mean 61 years	5–7 years	Employed (full or part-time); self-reported in telephone interview	67% employed
Dahl et al., 2015 [33]	Norway	Prospective survey Source: 14 urology clinics	*N* = 330; 100% prostate cancer; 100% male; age not reported	3 years	Working (full or part-time); self-reported on postal questionnaire	93% working
Hamood et al., 2018 [34]	Israel	Cross-sectional survey Source: Health insurance fund	*N* = 206; 100% breast cancer; 100% female; mean 49 years	3–14 years (mean 8.5 years)	Working (full or part-time); self-reported on questionnaire	67% working
Jagsi et al., 2014 [35]	USA, California, Michigan	Prospective survey Source: Population-based cancer registries	*N* = 746; 100% breast cancer; 100% female; mean 50 years	4 years	No longer working; self-reported on postal questionnaire	32% no longer working
Jeon, 2016 [36]	Canada	Prospective, linkage of cancer cases and non-cancer comparators Source: Administrative data	*N* = 2597; 26% breast, 11% cervical, 9% colorectal, 8% prostate; 63% female; mean 48 years *N* = 82,183 non-cancer comparators; 63% female, mean 48 years	3 years	Working^b^ from national statistics	85% of survivors working vs 94% of non-cancer comparison group
Johnsson et al., 2007 [37]	Sweden	Observational study, nested in prospective RCT Source: Five hospitals	*N* = 222 and 204; 100% breast cancer; 100% female; mean 47 years	2 and 3 years	Return to work; self-reported questionnaire	2 years: 84% returned to work 3 years: 86% returned to work
Kiserud et al., 2016 [38]	Norway	Cross-sectional survey Source: Four oncology departments	*N* = 265; 100% lymphoma; 40% female; mean 42 years	12 years	Employed^c^; self-reported by postal questionnaire	56% employed
Landeiro et al., 2018 [39]	Brazil	Prospective survey Source: single clinical center	*N* = 111; 100% breast cancer; 100% female; age not reported	2 years	Working (full-time or part-time); self-reported by telephone interview	60% working
Maunsell et al., 2004 [40]; Drolet et al., 2005a [41]; Drolet et al., 2005b [42]	Canada, Quebec	Cross-sectional survey of survivors and cancer-free controls recruited via provincial healthcare files Source: Population-based cancer registry	*N* = 646; 100% breast cancer; 100% female; mean 47 years Controls: *N* = 890; 1000% female, mean 45 years	3 years	Unemployed; self-reported by telephone interview	21% of survivors unemployed vs 15% of controls
Mols et al., 2009 [43]	Netherlands	Cross-sectional survey Source: Population-based cancer registry	*N* = 403; 25% prostate; 15% endometrial; 25% Hodgkin’s lymphoma; 35% non-Hodgkin’s; 40% female; mean 53 years	8.5 years	Working^d^; self-reported postal questionnaire	66% working
Paraponaris et al., 2010 [44]; Marino et al., 2013 [45]	France	Cross-sectional survey Source: National Health Insurance Fund	*N* = 1424; 41% breast; 5% prostate; 12% other urogenital; 32% other; 65% female; mean 47 years	2 years	Working; self-reported by telephone interview	66% working
Pearce et al., 2014 [46]	Ireland	Cross-sectional survey Source: Population-based cancer registry	*N* = 264; 32% larynx, 23% pharynx, 45% other sites in head and neck; 29% female; mean 52 years	2, 3, 4 and 5 years	Working; self-reported by postal questionnaire	2 years: 64% working 3 years: 68% working 4 years: 68% working 5 years: 68% working
Sanchez et al., 2004 [47]	USA, California	Cross-sectional survey Source: Two population-based cancer registries	*N* = 200; 100% colorectal; 54% female; mean 49 years	5 years	Employed; Self-reported by postal questionnaire	71% employed
Short et al., 2005 [48]; Farley Short et al., 2008 [49]; Moran et al., 2011 [50]	USA, Pennsylvania and Maryland	Cross-sectional interview with 1 year follow-up, and non-cancer comparator populations Source: Hospital tumor registries, and panel/labor market surveys^e^	*N* = 1433 and 1511; 31% breast, 8% prostate, 7% colorectal, 54% other sites; 64% female; mean 49 years Non-cancer comparators: *N* = 4141 (aged 28–54) and 3903 (aged 55–65)	2.5 years and 3.5 years	Return to work; self-reported by telephone interview	2.5 years: 81%^f^ returned to work 3.5 years: 84%^f^ returned to work
Tevaarwerk et al., 2013 [51]	United States, Wisconsin	Cross-sectional survey Source: 38 institutions	*N* = 225; 75% breast, 14% colorectal, 4% prostate, 7% lung; 84% female; mean 48 years	> 2 years (on average 4 years)	Working (full or part-time); self -reported	83% working
Tison et al., 2016 [52]; Alleaume et al. 2018 [53]	France	Cross-sectional survey with comparators Source: Three sickness funds and labor market survey (comparators)	2 years: *N* = 2055; various diagnoses; 59% female; mean 56 years; Non-cancer comparators: *N* = 2055; 52% female; mean 39 years 5 years: *N* = 969; 58% breast cancer, thyroid 10%, lung 7%; 82% female; 18–54 years at diagnosis	2 years 5 years	Employed; telephone survey or postal questionnaire (survivors) or face-to-face interview (comparators)	2 years: salaried individuals: 79% survivors versus 94% controls 2 years: self-employed: 86% survivors versus 96% controls 5 years: 82% cancer survivors
Van den Brink et al., 2007 [54]	Netherlands	Observational study nested within prospective RCT Source: 84 hospitals	*N* = 238; 100% rectal; 51% female; mean 52 years	2 years	Paid labor resumption; self-reported by questionnaire	70% paid labor resumption (55% completely; 15% partially)
Vartanian et al., 2006 [55]	Brazil	Cross-sectional survey Source: Single hospital	*N* = 301; oral cavity 53%, oropharynx 18%, larynx 26%, hypopharynx 3%; 22% female; median 52 years	> 2 years (on average 10 years)	Unable to work^g^; self-reported in face-to-face interview	33% unable to work
Verdonck-de Leeuw et al. 2010 [56]	Netherlands	Cross-sectional survey Source: Single hospital	*N* = 53; oral cavity/oropharynx 37%, larynx 34%, nasopharynx 18%, other head and neck site 12%; female 36%; median 59 years	> 2 years (on average 4 years)	Return to work; self-reported by postal questionnaire	83% returned to work

^a^Average was calculated if only range was given in article

^b^Inferred from non-zero earnings

^c^Including those on sick leave

^d^Non-cancer comparator population not included in initial paper. Analysis in subsequent papers was stratified by age and included comparators from different surveys

^e^Projected by life table analysis

^f^Did not stop working or retire

^g^Lost job or retired

### Study characteristics

Of the 21 studies, six were undertaken in the USA [29, 31, 35, 47, 48, 51], three in the Netherlands [43, 54, 56], two each in Brazil [39, 55], Canada [36, 40], France [44, 52], and Norway [33, 38], and one each in Ireland [46], Israel [34], Sweden [37], and UK [28]. Fourteen studies were cross-sectional [28, 31, 34, 38, 40, 43, 44, 46–48, 51, 52, 55, 56], five were prospective [29, 33, 35, 36, 39], and two contained both cross-sectional and prospective elements [37, 54]. Four studies included external comparison groups: one recruited controls matched to cases [40], two selected comparison populations from existing panel or labor market surveys [48, 52], and one used administrative data to identify the population without cancer [36]. Eleven studies used a population-based cancer registry [28, 31, 35, 40, 43, 46, 47] or administrative data [34, 36, 44, 52] as the sampling frame for survivors, with the other ten studies recruiting from hospital or clinical sources [29, 33, 37–39, 48, 51, 54–56]. In eight studies, survivors of a variety of cancers were included [28, 31, 36, 43, 44, 48, 51, 52]; six studies included only breast cancer survivors [29, 34, 35, 37, 39, 40]; three included head and neck cancers only (albeit at multiple sites within the head and neck) [46, 55, 56]; two included colorectal cancers only [47, 54]; one included hematological cancers only [38]; and one included prostate cancer only [33].

Sample sizes ranged from 53 to 2597, with 14,207 survivors included in total. The mean age of survivors varied from 42 to 61 years. Work retention was described in terms of working in 16 studies (“working” in 10 studies [28, 29, 33, 34, 36, 39, 43, 44, 46, 51]; “returned to work” in three [37, 48, 56]; “paid labour resumption” in one [54]; “no longer working” in one [35]; “unable to work” in one [55]) and in terms of employment in five studies (“employed” in four studies [31, 38, 47, 52]; and “unemployed” in one [40]).

Work outcomes were assessed by self-report in 20 studies and from administrative data in one study [36]. The time at which work retention was assessed ranged from 2 to 14 years post-diagnosis. Five studies (one prospective [29], three cross-sectional [46, 48, 52], and one mixed [37]) reported work retention at multiple time points.

### Quality assessment

Of the non-comparative studies, for which the maximum possible score was 16, ten scored ≤ 8 [28, 34, 38, 43, 44, 46, 47, 51, 55, 56] and seven scored 9 or more [29, 31, 33, 35, 37, 39, 54] (Supplementary Table S2). The four studies with a comparison population scored in the range 12–15 out of a maximum score of 24 [36, 40, 48, 52]. As a result, no studies scored as high quality mainly due to lack of non-cancer control groups. Across studies, the areas where studies scored poorly were lack of prospective data collection, unclear endpoints, failure to report a priori sample size calculation, and failure to report loss to follow-up.

### Workforce retention among long-term survivors

**Fig. 2:**
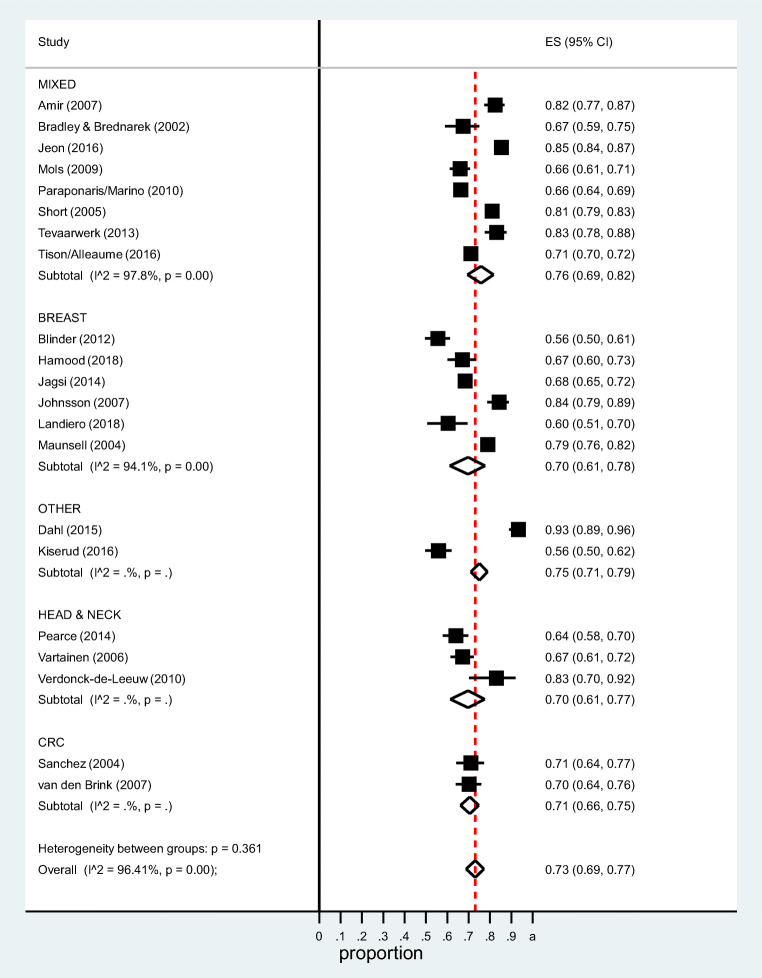
Proportion of survivors who have returned to work 2+ years post-diagnosis by cancer site

The pooled estimate of the proportion of survivors working at ≥ 2 years post-diagnosis was 0.73 (95%CI 0.69–0.77) (Fig. 2). Heterogeneity was significant (*I*^2^ = 96.4%). In the sensitivity analysis, in which the final (rather than earliest) time-point was included for the five studies which reported work retention at multiple time-points [30, 37, 46, 48, 52], the pooled estimate was 0.75 (95%CI 0.70–0.79, *I*^2^ = 96.0%).

The proportion working at different time-points after diagnosis was as follows: 2–2.9 years (reported in seven studies): 0.72 (95%CI 0.66–0.77); 3–3.9 years (8 studies): 0.80 (95%CI 0.74–0.86); 4–4.9 years (4 studies): 0.75 (95%CI 0.67–0.83); 5–5.9 years (4 studies): 0.74 (95%CI 0.66–0.81); 6+ years (5 studies): 0.65 (95%CI 0.60–0.69).

**Fig. 3:**
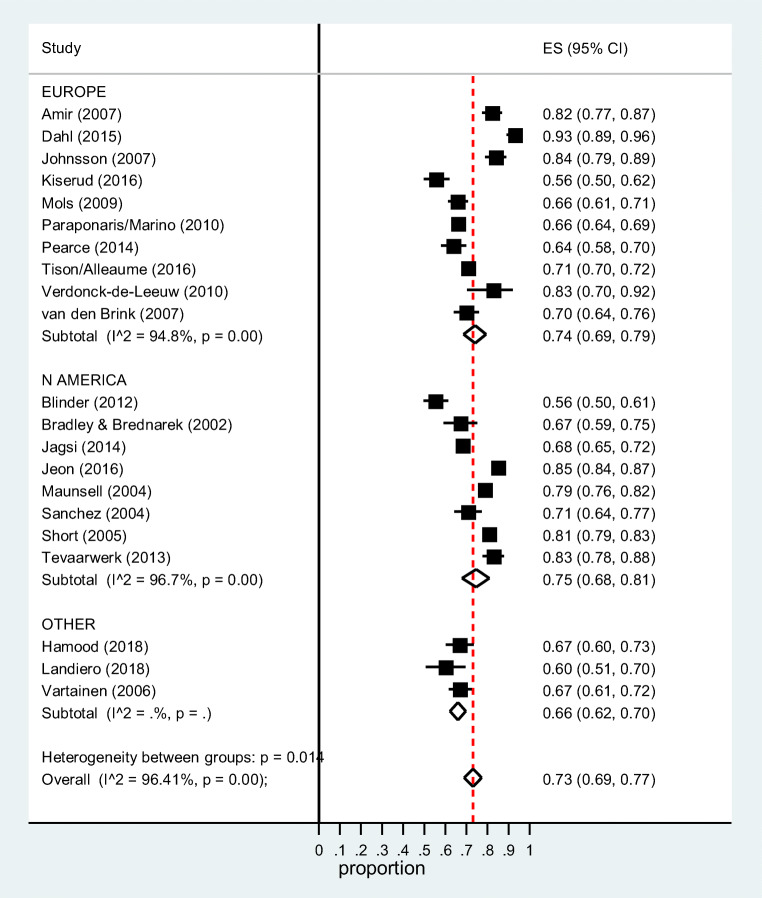
Proportion of cancer survivors who have returned to work 2+ years post-diagnosis by geographical area

Figure 2 shows that there was no significant difference in the pooled estimate between subgroups defined by cancer site (mixed sites: 0.76 (95%CI 0.69–0.82); breast: 0.70 (95%CI 0.61–0.78); head and neck: 0.70 (95%CI 0.61–0.77); colorectal: 0.71 (95%CI 0.66–0.75); and other individual sites: 0.75 (95%CI 0.71–0.79); subgroup heterogeneity Chi-square = 4.34, 4 *df*, *P* = 0.36). By geographical area (Fig. 3), the pooled estimates were Europe 0.74 (95%CI 0.69–0.79), North America 0.75 (95%CI 0.68–0.81), and elsewhere 0.66 (95%CI 0.62–0.70). There was no difference in the pooled estimate by study design (cross-sectional: 0.72 (95%CI 0.68–0.76); prospective: 0.75 (95%CI 0.65–0.84); subgroup heterogeneity Chi-square = 0.37, 1 *df*, *P* = 0.54) or data source (population-based/administrative: 0.72 (95%CI 0.67–0.77); clinical: 0.74 (95%CI 0.66–0.82); subgroup heterogeneity Chi-square = 0.21, 1 *df*, *P* = 0.65) (Supplementary Figures S1 and S2).

### Studies with non-cancer comparators

The five articles describing the four studies which included comparison groups reported lower long-term work retention among survivors than comparators [36, 40, 49, 50, 52]. In Canada, Maunsell et al. found that the relative risk of unemployment at 3 years was significantly higher among survivors (RR = 1.29, 95%CI 1.05–1.59) [40]. Also, in Canada, Jeon et al. reported that 85% of survivors were working at 25–47-month post-diagnosis compared to 94% of comparators [36]. In France, compared to matched comparators, the percentage of survivors who were employed at 2-years post-diagnosis was lower among both salaried (79% vs 94%) and self-employed (86% vs 96%) individuals [52]. In the USA, the employment rate at 2–6-years post-diagnosis was 7–8% lower for survivors aged 25–54 than age-matched comparators [50] and 4% lower for survivors aged 55–65 than similarly aged comparators [49].

### Risk factors for work retention among long-term survivors

Seven studies investigated patient-related, clinical, and/or work-related risk factors for work retention among long-term cancer survivors and analyzed them in a multivariate analysis [28, 29, 35, 39, 40, 52, 55] (Table 2).

**Table 2:** Risk factors for work retention among longer-term cancer survivors

Author and year	Risk factors assessed	Results^a^
Amir et al., 2007 [28]	• Patient-related: gender, deprivation • Clinical: surgery • Work-related: length of sick leave	Longer sick leave (OR = 1.68, 1.2–2.3) and absence of surgery (OR = 0.28, 0.08–0.9) were significantly associated with working 3 years after diagnosis
Blinder et al., 2012 [29]; Blinder et al., 2013 [30]	• Patient-related: age, race/ethnicity, birthplace, household income, adequate financial resources, marital status, children living at home, seniors living at home, education, acculturation, social support • Clinical: comorbid conditions, stage at diagnosis, type of surgery, breast reconstruction, axilliary node dissection, chemotherapy, radiotherapy, endocrine therapy • Work-related: job type, full/part-time work at diagnosis	Presence of comorbid conditions (OR = 0.25, 0.08–0.7) was significantly associated with not returning to work 3–5 years postdiagnosis
Jagsi et al., 2014 [35]	• Patient-related: age, race, education, family income, marital status, area of residence, family income • Clinical: comorbidities, stage at diagnosis, type of surgery, chemotherapy, radiotherapy • Work-related: full/part time work at diagnosis, employment support (sick leave/flexible schedule)	Older age at diagnosis (≥ 56 vs < 46: OR = 1.42, 1.03–1.9), receipt of chemotherapy (OR = 1.42, 1.03–1.98), comorbidities (≥ 2 vs none: OR = 2.16, 1.6–2.9), and lack of work adjustments (none vs sick leave and/or flexible schedule vs: OR = 1.33, 1.1–1.6) were significantly associated with unemployment
Landeiro et al., 2018 [39]	• Patient-related: education, age, changes in marital status, • Clinical: health status, weight gain, depression, pain, lymphedema, breast conserving surgery, breast reconstruction, axillary dissection, chemotherapy, radiotherapy, endocrine therapy, anti-HER2 therapy, quality of life • Work-related: changes in income, work adjustment, employer discrimination, employer support	Higher household income (OR = 16.6, 1.8–155), work adjustments (OR 37.6, 3.31–427), breast conserving surgery (OR 9.8, 2.0–47), not having depression (OR 14.3, 1.6–100), and not having endocrine therapy (OR 9.1, 1.3–50) were significantly associated with working at 2 years post-diagnosis
Maunsell et al., 2004 [40]; Drolet et al., 2005a [41]; Drolet et al., 2005b [42]	• Patient-related: age, living with partner, children, education, personal income • Clinical: disease status since diagnosis (disease-free vs recurrence/contralateral breast cancer); radiotherapy, chemotherapy, hormone therapy, affected nodes • Work-related: union member, experience in job, type of job, hours per week, value of work	Significant predictors of not working at 3 years were: older age (50–59 vs 18–39 OR = 4.62, 2.2–9.5), lower personal income (< $20,000 vs ≥ $50,000 OR = 3.18, 1.6–6.3), new cancer event (OR = 2.14, 1.5–3.1), union membership (union membership yes vs no OR = 1.88, 1.3–2.7; self-employed vs not union member OR = 0.60, 0.3–1.05), and value of work since diagnosis (decreased vs increased: OR = 1.83, 1.1–3.0)
Tison et al., 2016 [52]; Alleaume et al. 2018 [53]	• Patient-related: marital status, gender, age, dependent children • Clinical: cancer prognosis, adverse cancer event, chemotherapy, radiotherapy, comorbidities, mental health, chronic neuropathic pain • Work-related: employment sector at diagnosis, socio-professional status, wages at diagnosis, full-time/part-time at diagnosis, type of employment contract, self-employed versus employee, business sector	Older age, not having children, and poor cancer prognosis, were significantly related to not working at 2 years after cancer diagnosis. Age 18–39 (OR 1.69, 1.00–2.9) or age 50–54 (OR 1.65, 1.06–2.6), not having children (OR 2.1, 1.3–3.4), poor cancer prognosis (OR 3.6, 1.6–8.2), adverse cancer event (OR 2.1, 1.3–3.3), chemotherapy (OR1.6, 1.1–2.4), comorbidities (OR 2.0, 1.2–3.4), mental health (OR 0.96, 0.95–0.98), chronic neuropathic pain (OR 2.6, 1.7–3.9), private sector (OR 2.5, 1.5–4.3), execution function (OR 2.2, 1.4–3.2), and higher wages at diagnosis (OR 1.01, 0.99–1.03) were significantly related to leaving employment at 5 years after cancer diagnosis
Vartanian et al. 2006 [55]	• Patient-related: gender, age, alcohol use, education, pain, quality-of-life score • Clinical: cancer site, stage, treatment, permanent tracheostomy	More advance stage (VI vs I OR = 3.5, 1.5–8.1), alcohol use before treatment (OR = 2.6, 1.3–5.2), and lower education (high school or college vs illiterate OR = 0.2, 0.5–0.8) were significantly associated with being unable to work > 2 years post-diagnosis

^a^Only results significant in multivariable analyses are reported

Of the patient-related factors, older age [35, 40, 52] and lower income at diagnosis [39, 40, 52] were significantly associated with not returning to work in multivariate analyses in three studies. The clinical factors receiving chemotherapy [35, 52], having comorbidities [30, 35, 52], having a new cancer event [40, 52], having a poor prognosis [52, 55] or depression [39, 52], and the work-related factor lack of work adjustments [35, 39] were associated in multivariate analyses with not returning to work.

### Other work-related outcomes

Sixteen studies reported other work-related outcomes among survivors (Table 3). Of the nine studies which examined changes in working hours among survivors [28, 33, 34, 39, 40, 43, 46, 48, 52], six studies reported that 12–52% of survivors who had returned to work had reduced their working hours compared to before diagnosis [28, 39, 40, 43, 46, 52]. One study reported that survivors worked fewer hours than similarly aged people without cancer [48]. In three studies, the proportion of survivors working part-time had increased and/or the proportion working full-time had decreased [33, 34, 40].

**Table 3:** Other work-related outcomes among longer-term cancer survivors

Author and year	Work-related outcomes assessed	Results
Amir et al., 2007 [28]	• Change in working hours • Change in place of work • Perception of work	• 18% of survivors who took < 6 months sick leave, and 43% of those who took ≥ 18 months sick leave, changed their working hours compared to before diagnosis • 8% of survivors who had returned to work changed to a different place of work • 19% of survivors who returned to work reported that their overall working life had deteriorated due to cancer
Bradley and Bednarek, 2002 [31]; Bednarek and Bradley, 2005 [32]	• Change in work schedule	• 54% of survivors reduced their workload/working schedule at least once because of cancer
Dahl et al., 2015 [33]	• Reduced working hours • Influence of prostate cancer on working life	• 66% of survivors worked full-time at 3 years compared to 75% at diagnosis • 34% of survivors reported that prostate cancer had influence their working life to some/great extent. In multivariable analysis among men active in the workforce, adjuvant/salvage treatment, chronic fatigue, physical work and bother with urinary leakage were significantly associated with believing prostate cancer had influenced working life to some/great extent.
Hamood et al., 2018 [34]	• Change in working hours	• At a mean of 8.5 years post-diagnosis, 48% of survivors had changed from full-time to part-time employment. In multivariate analyses, immigration status (country of birth not Israel) was significantly associated with changing from full-time to part-time employment
Jagsi et al., 2014 [35]	• Seeking work	• At 4 years post-diagnosis, 39% of survivors who were not employed were actively looking for work
Jeon, 2016 [36]	• Income	• During 25–47 months post-diagnosis, survivors earned 9.0% less than comparators. The difference was greatest for those with cancers of low survival.
Kiserud et al., 2016 [38]	• Work changes due to cancer • Work ability	• 13% of survivors who returned to work reported work changes due to cancer • Work ability was higher among those working at survey than not working (mean = 7.3 vs 3.6); 11% of those working vs 59% of those not working had poor physical work ability; 6% of those working vs 33% of those not working had poor mental work ability; change in work ability was lower among those working than those not working
Landeiro et al., 2018 [39]	• Change in working hours • Income • Perceived employer discrimination	• Among survivors who returned to work, 12% decreased and 3% increased working hours • 21% reported a reduction in monthly income • 11% reported perceived employer discrimination
Maunsell et al., 2004 [40]; Drolet et al., 2005a [41]; Drolet et al., 2005b [42]	• Change in working hours • Change in job • Income • Sickness absence	• Among survivors employed at 3 years, hours worked per week in main/only and any second job were significantly lower than at diagnosis • 19% of survivors (20% of those disease-free and 13% of those not disease-free) vs 20% of comparators were employed in a different job than at diagnosis • At 3 years, the increase in the proportion who earned $30,000+ per annum (compared to at diagnosis) was similar in survivors and comparators • In the third year from diagnosis, 23% of survivors were absent from work for ≥ 4 weeks vs 19% of comparators. Average duration of absence was longer in survivors who were not disease free, compared to those who were disease free (4.1 weeks vs 2.1 weeks).
Mols et al., 2009 [43]	• Change in working hours	• At survey, 17% of survivors worked fewer hours than at diagnosis
Paraponaris et al., 2010 [44]; Marino et al., 2013 [45]	• Sickness absence	• 20% of survivors who were employed at diagnosis and at 2 years took no sick leave
Pearce et al., 2013 [46]	• Change in working hours	• Among survivors who returned to work, 52% reduced and 3% increased working hours compared to at diagnosis
Sanchez et al., 2004 [47]	• Sickness absence	• Of survivors who resumed working, 36% returned after ≥ 60 days absence. In multivariate analyses, receipt of chemotherapy was significantly related to returning after 60 days
Short et al., 2005 [48]; Farley Short et al., 2008 [49]; Moran et al., 2011 [50]	• Hours worked	• At 2–6 years post-diagnosis, female survivors aged 28–54 worked 3–4 hours less per week than similarly-aged females in comparison population; male survivors aged 28–54 worked 5–6 hours less than similarly-aged males in comparison population. Female survivors aged 55–65 worked 3–4 hours less per week than similarly-aged females in comparison population; male survivors aged 55–65 worked 3.5–5 hours less than similarly-aged males in comparison population
Tison et al., 2016 [52]; Alleaume et al., 2018 [53]	• Change in working hours	• Of survivors who had returned to work at 5 years, 32% had reduced working hours compared to diagnosis. In multivariate analysis, wages at diagnosis, sector of employment at diagnosis, chemotherapy, mental health score and chronic neuropathic pain were significantly associated with reduced working hours at 5 years
Verdonck-de Leeuw et al., 2010 [56]	• Change in work	• Of survivors who resumed working, 36% had changed work (i.e. returned to adapted work or to other work).

Five studies reported on other changes in survivors’ work situations. There was no difference in the proportion of breast cancer survivors (19%) who had changed job at 3 years compared to non-cancer comparators [40]. Another study of a mixed group of survivors reported that 8% had changed employer at 3 years [28]. Three studies reported that a proportion of survivors (13–55%) had had to reduce workload, change their working schedule, or make adaptations due to cancer [31, 38, 56].

Three studies described income post-diagnosis [36, 39, 40]. In one, during 25–47-month post-diagnosis, survivors earned 9% less than comparators [36] and, in another, 21% of survivors reported reduced monthly income [39]. In contrast, at 3 years, the increase in the proportion who earned ≥ $30,000 per annum (compared to at diagnosis) was similar in survivors and comparators [40].

## Discussion

### Summary of main findings

This systematic review indicates that 73% of long-term cancer survivors who were working at diagnosis return to work and that long-term survivors are less likely to be working than people without cancer. However, there is significant heterogeneity in estimates of work retention between studies. Prognostic factors for not returning to work among long-term survivors include older age, lower income at diagnosis, comorbidities, receipt of chemotherapy, and lack of work adjustments, but these have been investigated in relatively few studies. In terms of other outcomes, a proportion of long-term survivors reduce their working hours compared to at diagnosis, and some make other work-related changes; they may also have reductions in income. However, these outcomes have been reported in few studies.

### Interpretation of results

Our pooled estimate of the prevalence of work retention in long-term survivors is slightly higher than estimates of return to work from previous reviews which largely included studies of shorter-term survivors (Spelten et al., 62% [18]; Mehnert, 64% [19]). For the current review, only studies in which all survivors were working at diagnosis were eligible for inclusion; this was not a requirement in previous reviews and could explain the apparently higher rate of work resumption observed here (since not working at diagnosis is a predictor of not working after cancer [44, 57]). Although some studies suggest that a longer period of work absence after a cancer diagnosis is associated with reduced likelihood of work resumption [28, 45], there is also evidence that rates of sickness absence post-cancer decrease over time and a proportion of those who are absent long-term eventually return to work [46, 58]. Thus, the higher rate of return to work in long-term survivor may be real.

To shed further light on this, we sought to investigate the temporal trajectory of work retention in long-term survivors. However, only five studies reported work resumption at more than one time-point (and three of these had a cross-sectional design) [29, 37, 46, 48, 52], and information on work retention at 6 or more years post-diagnosis was only available from five studies which reported outcomes at a heterogeneous range of follow-up times (e.g., 5–7 years, 12 years, > 2 years) [31, 34, 38, 43, 55]. Nonetheless, the meta-analysis suggested that there may be a modest trend in work retention—higher in years 3–3.9 than years 2–2.9 followed by a modest decline in later periods. This later decline is consistent with a recent Japanese study which showed that, among male cancer survivors, the rate of work continuation after return to work decreased steadily over time and that, on average, survivors continued working for only 4.5 years after work resumption [59].

The decline in work participation over time could reflect people dropping out of the workforce due to diagnosis of a second primary cancer or other cancer-related symptoms or late effects. Survivors who have returned to work can experience a range of physical or psychological after-effects which adversely impact their work ability or functioning [60, 61]. In addition, cancer-related symptoms, such as fatigue, pain, and depression, have been associated with leaving the workforce after cancer, albeit mainly in shorter-term survivors [62–64]. Alternatively, the decline may simply reflect ageing and people reaching retirement age or opting for early retirement. While a significant proportion of cancer survivors want to retire early [65], and there is an excess risk of early retirement among survivors [66], some of those who do retire feel that they were forced into this rather than it being a free choice [32]. Further research is needed to clarify long-term temporal trajectories of work retention (and related outcomes, such as early retirement) among cancer survivors, and the factors influencing survivors’ decisions to leave the workforce after initially resuming work.

We found a suggestion of geographical variation in work retention after cancer, with a lower prevalence in studies conducted outside North America and Europe. However, there was significant within-group heterogeneity so it is likely that the *P* value from the test of between-group heterogeneity is too small [67]. Moreover, only three studies were included from outside Europe and North America, two from Brazil and one from Israel, and the largest of these included only 301 survivors [34, 39, 55]. Our rationale for grouping countries was that there is legislation intended to protect cancer survivors against discrimination at work in place in much of Europe [68] and North America (e.g., Americans with Disabilities Act [69]), but this may not be the situation elsewhere. Given this, it was striking that we found almost identical prevalence of work resumption in the studies from Europe and North America. This is consistent with conclusions from a 2009 meta-analysis of risk of unemployment in cancer survivors which reported no significant difference between Europe and the USA once cancer site, age, and background employment rate had been accounted for [2]. However, it is worth noting that the prevalences reported here from the European and North American studies varied widely (Europe: range 56% [38] to 93% [33]; North America: 56% [29] to 85% [36]). This indicates the need for further large-scale studies of long-term survivors in all settings.

The prognostic factors for work retention among long-term survivors identified here are broadly consistent with those reported in reviews which have mainly considered short-term work outcomes [18, 19, 70]. For example, in reviews of prognostic factors for return to work following breast or colorectal cancer, older age, receipt of chemotherapy, and presence of comorbidities were identified as inhibiting factors [71, 72], which we also found. Several other prognostic factors identified here—such as lack of work adjustments, self-employment, perceived value of work, and not having children—were seen in single studies and require confirmation.

### Strengths and limitations

This is the first review to focus on long-term survivors, a group of growing size. We followed systematic review guidelines in conduct and reporting (PRISMA) [23] and used a valid and reliable tool to assess quality (MINORS) [27]. We minimized the possibility of missing relevant articles by searching multiple databases using terms designed to be sensitive and by reviewing reference lists of included papers and other reviews. To maximize comparability of estimates of prevalence of work retention across studies, we considered studies eligible only if all survivors were in paid work at the time of diagnosis. Despite this, there was heterogeneity in the estimates of work retention observed and it is likely that this was driven by the heterogeneity in study design and conduct. For example, authors used different terms for work retention (e.g., working, employed) but failed to define precisely what these meant (e.g., whether the “employed” group included people who were still on sick leave); most failed to state whether both full and part-time work was considered as working post-diagnosis; and most did not indicate whether they excluded some groups of survivors from follow-up (e.g., those with recurrent disease). All of these issues could have a significant impact on the estimate of work retention.

In addition, the quality appraisal indicated that none of the studies would be considered high quality. It is well recognized that studies of workforce participation after cancer should include non-cancer comparators to allow for the effect of cancer on workforce participation and general labor market trends to be distinguished [73]. Despite this, surprisingly, few studies (only 4) included non-cancer comparators. This contrasts with the 2009 systematic review of unemployment in cancer survivors which included 26 studies, all of which had non-cancer comparators [2]. Studies in the current review scored poorly in terms of lack of prospective design, failure to report a priori sample size calculations and failure to report loss to follow-up. In addition, work retention was self-reported in 20 of the 21 studies, using a variety of data collection methods and instruments/questions; none of these instruments/questions appeared to have been rigorously validated. Most studies were small—only six included more than 500 survivors [35, 36, 40, 44, 48, 52]. Eight included a mixed group of cancer survivors (and insufficient numbers to allow site-specific analyses) [28, 31, 36, 43, 44, 46, 48, 51, 52], even though cancer site is likely to be a significant prognostic factor for work-related outcomes [59, 70].

Considering the evidence-base as a whole, this review indicates that important gaps remain around work retention in long-term cancer survivors. Little is known about patterns and predictors of long-term work retention in most countries beyond North America and selected European populations. System-level factors (e.g., social welfare provisions, insurance, legal provisions) are likely to be important influences on work outcomes among cancer survivors [74], but have not been investigated as influences among long-term survivors. Little is known about most cancers, other than breast (most of the studies of mixed cancer sites were dominated by breast cancer). Little is known about how work retention—and other work-related outcomes (such as income)—evolve over time in long-term survivors.

### Future directions: Research

High-quality, population-based, longitudinal studies, which include non-cancer comparators, are needed to fill the evidence gaps identified by this review and clarify the work retention trajectory in long-term cancer survivors. While studies involving primary data collection would be useful (as they allow collection of detailed data about work outcomes and prognostic factors), studies which involve linkage of administrative and health datasets would also be of considerable value (see, for example, Grinshpun [57], Jeon [36], Heinesen [75]). This review also indicates a clear need for harmonization of data and methods across the research community. In particular, there is an urgent need to develop standard instruments to assess work retention (and other work-related outcomes) which could be used internationally. The European CANWON network [76] has embarked on a consensus process to develop such a tool. Initiatives to pool patient-level data from studies in different settings could also be of value in understanding system-level drivers.

### Future directions: Practice

The findings of this review—particularly regarding the proportion of survivors who retain work long-term—are relevant for patients and patient advocacy groups, and for cancer clinicians, oncology nurses, general physicians, and occupational health care professionals who counsel and advise cancer patients. Professionals may also consider focussing support efforts on those subgroups of survivors most likely to have poor long-term work retention outcomes. The findings are also pertinent for the development and update of oncological guidelines on cancer survivorship care.

## Conclusions

This systematic review indicates that 73% of long-term cancer survivors who were working at diagnosis return to work, and that long-term survivors are less likely to be working than people without cancer. Prognostic factors for not returning to work among long-term survivors include older age, lower income at diagnosis, comorbidities, and receipt of chemotherapy, but these have been investigated in relatively few studies. High-quality, population-based, longitudinal studies, which include non-cancer comparators, are needed to fill the evidence gaps identified by this review and clarify the work retention trajectory in long-term cancer survivors.

### Electronic supplementary material

ESM 1 (DOCX 66 kb)

## Abbreviations

CANWON: CANcer and WOrk Network
CI: confidence interval
df: degrees of freedom
MINORS: Methodological Index for Non-Randomised Studies
PRISMA: Preferred Reporting Items for Systematic Reviews and Meta-Analyses
RR: relative risk
