# Supplementary material for: Long-term work retention after treatment for cancer: a systematic review and meta-analysis
Source: J Cancer Surviv. 2020 Mar 11;14(2):135–50. doi: 10.1007/s11764-020-00862-2 (PMC7182621; doi:10.1007/s11764-020-00862-2)
Supplement: Supplementary file 1 — (DOCX 66 kb) [file 11764_2020_862_MOESM1_ESM.docx]

**Supplementary Table S1: Search terms for review**

| (neoplasms [MeSH Terms] or cancer* [Text Word] or neoplasm* [Text Word] or carcinoma* [Text Word] or oncolog* [Text Word] or malignan* [Text Word] or tumor [Text Word] or tumour [Text Word] or tumors [Text Word] or tumours [Text Word] or radiotherapy [Text Word] or chemotherapy [Text Word]) AND |
| --- |
| (employment [MeSH Terms] or employment [Text Word] or unemployment [MeSH Terms] or unemployment [Text Word] or unemployed [Text Word] or retirement [Text Word] or “sick leave” [MeSH Terms] or “sick leave” [Text Word] or “Sickness absence” [Text Word] or absenteeism [MeSH Terms] or absenteeism [Text word] or “work” [MeSH Terms] or occupations [MeSH Terms] or “disability management” [Text word] or “rehabilitation, vocational” [MeSH Terms] or rehabilitation [MeSH Terms:NoExp] or “neoplasms/rehabilitation” [MeSH Terms] or vocational* [Text Word] or “work ability” [Text Word] or “work capacity” [Text Word] or “work activity” [Text Word] or “work disability” [Text Word] or “work rehabilitation” [Text Word] or “work status” [Text Word] or “work retention” [Text Word] or workability [Text Word] or employability [Text Word] or employable [Text Word] or employee* [Text Word] or presenteeism [Text Word] or job satisfaction [Text Word] or career mobility [Text Word] or career choice [Text Word] or “work productivity” [Text Word] or “work hours” [Text Word] or income [Text Word]) AND |
| (survivors or surviv*[tiab] or disease-free survival [Text Word] or long term [Text Word] or long-term [Text Word] or longer term [Text Word] or longer-term [Text Word]) |
| NOT ( “primary prevention” [MeSH Terms] or "Neoplasms/prevention and control"[MeSH Terms] or "Smoking/prevention and control"[MeSH] or “smoking cessation” [MeSH Terms] or “Smoking/adverse effects”[MeSH Terms] or “occupational exposure” [MeSH Terms] or occupational exposure [Text Word] or “occupational diseases” [MeSH Terms] or occupational risk factor [Text Word] or “protective clothing” [MeSH Terms] or “inhalation exposure” [MeSH Terms ] or exposure [Text Word] or exposed [Text Word] or body mass [tw] or tobacco [tw] or occupational vitiligo [Text Word] or "Antineoplastic Agents"[Mesh] or "Molecular Structure"[Mesh] or "Immunoconjugates"[Mesh] or "Mutagenesis"[Mesh] or "Apoptosis"[Mesh] or apoptosis [Text Word] or "Tumor Markers, Biological"[Mesh] or marker* [tw] or genet* [tw] or "Signal Transduction"[Mesh] or toxin [Text Word] or toxin* [Text Word] or toxic* [Text Word] or toxic [Text Word] or "Toxicology"[Mesh] or "Carcinogens, Environmental/adverse effects"[MeSH] or “Mass Screening” [MeSH Terms] or screening [tw] or “Palliative Care” [MeSH Terms] or “end of life” [tw] or palliative [tw] or “Neoplasm Metastasis” [MeSH Terms] or “Mortality” [MeSH Terms] or “aged, 80 and over” [MeSH Terms] or “terminal care” [MeSH Terms] or “geriatric assessment” [MeSH Terms] or childhood [tw] or “non-cancer” [tw] or “non-malignant” [tw] or “gene expression profiling” [MeSH Terms] or "Radiology/education" [MeSH Terms]) |
| NOT (animal[mh] NOT human[mh]) |

**Supplementary Table S2: Methodological quality assessment**

| **Author** | **Aim^a^** | **Rate^b^** | **Data^c^** | **Measure^d^** | **Bias^e^** | **Time^f^** | **Loss^g^** | **Size^h^** | **Controls^i^** | **Contemporary^j^** | **Equivalence^k^** | **Analysis^l^** | **Total^m^** | **Total^n^** |
| --- | --- | --- | --- | --- | --- | --- | --- | --- | --- | --- | --- | --- | --- | --- |
| Amir et al., 2007 [29] | 2 | 2 | 0 | 1 | 1 | 1 | 0 | 0 | - | - | - | - | 7 |  |
| Blinder et al., 2012 [30] | 2 | 2 | 0 | 2 | 1 | 1 | 0 | 1 | - | - | - | - | 9 |  |
| Bradley & Bednarek, 2002 [32] | 2 | 1 | 2 | 1 | 1 | 2 | 2 | 0 | - | - | - | - | 11 |  |
| Dahl et al., 2015 [34] | 2 | 0 | 2 | 2 | 1 | 1 | 1 | 0 | - | - | - | - | 9 |  |
| Hammood et al., 2018 [35] | 2 | 2 | 0 | 2 | 1 | 0 | 0 | 0 | - | - | - | - | 7 |  |
| Jagsi et al., 2014 [36] | 2 | 1 | 2 | 1 | 1 | 1 | 2 | 0 | - | - | - | - | 10 |  |
| Jeon, 2016 [37] | 2 | 2 | 1 | 0 | 2 | 0 | 1 | 0 | 1 | 2 | 2 | 2 |  | 15 |
| Johnsson et al., 2007 [38] | 2 | 2 | 2 | 2 | 1 | 1 | 1 | 0 | - | - | - | - | 11 |  |
| Kiserud et al., 2016 [39] | 2 | 2 | 0 | 1 | 1 | 0 | 0 | 0 | - | - | - | - | 6 |  |
| Landeiro et al., 2018 [40] | 2 | 2 | 2 | 1 | 1 | 2 | 2 | 0 | - | - | - | - | 12 |  |
| Maunsell et al., 2004 [41] | 2 | 1 | 0 | 1 | 1 | 1 | 0 | 0 | 2 | 2 | 2 | 2 |  | 14 |
| Mols et al., 2009 [44] | 2 | 2 | 0 | 0 | 1 | 0 | 0 | 0 | - | - | - | - | 5 |  |
| Paraponaris et al., 2010 [45] | 2 | 1 | 0 | 2 | 1 | 1 | 0 | 0 | - | - | - | - | 7 |  |
| Pearce et al., 2013 [47] | 2 | 1 | 1 | 0 | 1 | 2 | 0 | 0 | - | - | - | - | 7 |  |
| Sanchez et al., 2004 [48] | 2 | 2 | 0 | 1 | 1 | 1 | 0 | 0 | - | - | - | - | 7 |  |
| Short et al., 2008 [49] | 2 | 2 | 2 | 1 | 1 | 2 | 1 | 0 | 0 | 2 | 0 | 2 |  | 15 |
| Tevaarwerk et al., 2013 [52] | 2 | 0 | 0 | 0 | 1 | 0 | 0 | 0 | - | - | - | - | 3 |  |
| Tison et al., 2016 [53] | 2 | 0 | 0 | 2 | 1 | 2 | 0 | 0 | 1 | 2 | 0 | 2 |  | 12 |
| Van den Brink et al., 2007 [55] | 2 | 0 | 2 | 1 | 1 | 1 | 2 | 0 | - | - | - | - | 9 |  |
| Vartanian et al., 2006 [56] | 2 | 0 | 0 | 1 | 1 | 0 | 0 | 0 | - | - | - | - | 4 |  |
| Verdonck-de Leeuw et al., 2010 [57] | 2 | 1 | 0 | 0 | 1 | 0 | 0 | 0 | - | - | - | - | 4 |  |

Assessed with the adapted Methodological index for non-randomized studies (MINORS) [18] ^a^ Clearly stated aim (0,1,2 points); ^b^ Inclusion criteria and response rate (0,1,2); ^c^ Prospective collection of data (0,1,2); ^d^ Inclusion of employment measure (0,1,2); ^e^ Unbiased assessment of study endpoints (0,1,2); ^f^ Follow-up time appropriate (0,1,2); ^g^ Loss to follow-up (0,1,2); ^h^ Prospective calculation of the study size (0,1, 2); ^i^ Adequate control group (0,1,2); ^j^ Contemporary comparison of groups (0,2); ^k^ Baseline equivalence of groups (0,1,2); ^l^ Adequate statistical analysis (0,1,2); ^m^ Non-comparative studies: Total: minimum=0, maximum = 16 points; ^n^ Studies with comparison population: Total: minimum=0, maximum = 24 points.
